# Advanced Application of Polymer Nanocarriers in Delivery of Active Ingredients from Traditional Chinese Medicines

**DOI:** 10.3390/molecules29153520

**Published:** 2024-07-26

**Authors:** Zhiyuan Zhai, Jianda Niu, Liguo Xu, Jinbao Xu

**Affiliations:** 1 School of Materials and Energy, Guangdong University of Technology, Guangzhou 510006, China; 2 College of Light Chemical Industry and Materials Engineering, Shunde Polytechnic, Foshan 528333, China

**Keywords:** Traditional Chinese Medicines, drug delivery, polymer micelles, polymer vesicles, polymer hydrogels, polymer drug conjugates

## Abstract

Active ingredients from Traditional Chinese Medicines (TCMs) have been a cornerstone of healthcare for millennia, offering a rich source of bioactive compounds with therapeutic potential. However, the clinical application of TCMs is often limited by challenges such as poor solubility, low bioavailability, and variable pharmacokinetics. To address these issues, the development of advanced polymer nanocarriers has emerged as a promising strategy for the delivery of TCMs. This review focuses on the introduction of common active ingredients from TCMs and the recent advancements in the design and application of polymer nanocarriers for enhancing the efficacy and safety of TCMs. We begin by discussing the unique properties of TCMs and the inherent challenges associated with their delivery. We then delve into the types of polymeric nanocarriers, including polymer micelles, polymer vesicles, polymer hydrogels, and polymer drug conjugates, highlighting their application in the delivery of active ingredients from TCMs. The main body of the review presents a comprehensive analysis of the state-of-the-art nanocarrier systems and introduces the impact of these nanocarriers on the solubility, stability, and bioavailability of TCM components. On the basis of this, we provide an outlook on the future directions of polymer nanocarriers in TCM delivery. This review underscores the transformative potential of polymer nanocarriers in revolutionizing TCM delivery, offering a pathway to harness the full therapeutic potential of TCMs while ensuring safety and efficacy in a modern medical context.

## 1. Introduction

Traditional Chinese Medicines (TCMs) offer a treasure trove of therapeutic agents for a myriad of health conditions and significantly contribute to the medical field. Active ingredients from TCMs, which harness ingredients from botanical, zoological, and mineral sources, are gaining global recognition for their health benefits and therapeutic potential [1,2,3]. However, the widespread application of TCMs is impeded by challenges such as variable quality, low solubility, stability issues, potential side effects, and a lack of precise targeting mechanisms [4]. Despite these hurdles, the therapeutic efficacy of TCMs is largely attributed to their bioactive molecules, including carboxyl, alcohol, phenol, and amine groups. These components, which constitute the majority of TCMs, exhibit pharmacological properties that are responsible for their distinct medicinal actions, such as addressing compatibility concerns, synergistic interactions among multiple constituents, and engaging multiple biological targets [5,6,7]. Therefore, a thorough exploration and strategic application of these active molecules could unlock new horizons for TCMs, solidifying their position as a robust and viable therapeutic alternative in contemporary medicine.

Recent advancements in nanocarrier-based drug delivery research present significant benefits in addressing the inherent limitations of TCMs, including their low bioavailability, limited aqueous solubility, and variable stability. The escalating publication of studies on the nanocarrier-mediated delivery of active molecules in recent years underscores an evolving appreciation of the capabilities of nanocarriers [8,9,10]. Nanocarriers enhance the bioavailability and targeting precision of TCMs while concurrently reducing adverse effects. The enhanced permeability and retention effect inherent to nanocarriers facilitates passive targeting, allowing for more efficient delivery to disease sites [11]. Furthermore, active targeting can be realized through nanocarriers equipped with ligands that bind to specific receptors, thereby amplifying the targeting efficacy of TCMs. These systems can also extend drug release periods, enabling controlled release profiles and diminishing the accumulation of toxins, which optimizes therapeutic outcomes. Additionally, nanocarriers enhance the water solubility and stability of hydrophobic TCM components, thereby improving their overall bioavailability and efficiency [12,13,14].

A diverse array of nanocarriers has been leveraged to augment the therapeutic profiles of TCMs within the biomedical domain, encompassing lipid-based systems such as ethosomes and transfersomes, polymer nanocarriers, and inorganic nanoparticles [15,16,17]. Among these, polymer nanocarriers have garnered the most extensive research attention, attributed to their capacity to enhance bioavailability and ensure stability, offer versatile formulation options, facilitate targeted delivery and tissue penetration, and enable controlled release mechanisms [18,19,20]. This review delves into the physicochemical characteristics of the principal bioactive constituents found in TCMs—terpenoids, flavonoids, alkaloids, and polyphenols—and endeavors to pave the way for an exhaustive examination of the cutting-edge applications of polymer nanocarriers in TCM delivery. We will explore the delivery of these valuable compounds and demonstrate how polymer nanocarriers can effectively achieve this goal. By undertaking this approach, this review lays the groundwork for an in-depth assessment of the capacity of polymer nanocarriers to unlock the comprehensive therapeutic potential of TCMs, positioning their inherent healing properties at the forefront of modern medical applications.

## 2. Active Ingredients from TCMs

### 2.1. Terpenoids

**Figure 1:**
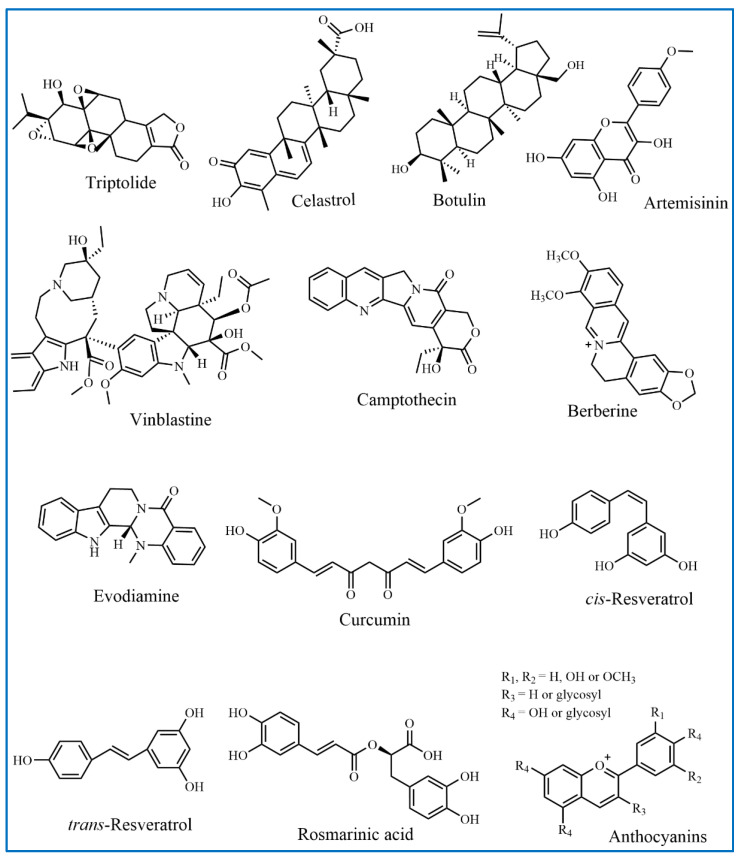
Chemical structures of active ingredients from Traditional Chinese Medicines.

Terpenoids, also known as isoprenoids, constitute a vast family of natural products and represent one of the most abundant groups of organic compounds found in nature, exemplified by compounds such as triptolide, celastrol, botulin, and artemisinin (Figure 1). These structurally varied compounds originate from the biosynthesis of simple precursors, isopentenyl diphosphate and dimethylallyl diphosphate, through the meandering routes of the isoprenoid pathway—a sophisticated metabolic cascade involving multiple enzymatic reactions [21,22]. Terpenoids are omnipresent in the plant kingdom, yet they are also detected in fungi, bacteria, and animals and they are celebrated for their wide array of pharmacological activities and biological roles. Research has illuminated the diverse biological effects of terpenoids, including anti-inflammatory, antimicrobial, anticancer, and antiparasitic actions, endowing them with significant appeal for the formulation of innovative therapeutic agents [23,24,25,26].

### 2.2. Flavonoids

Flavonoids represent the most common secondary metabolites in plants, are a result of natural selection, and are predominantly found in various plant parts, especially petals, leaves, and fruits [27]. Characterized by a fundamental 2-phenyl chromone (C6-C3-C6) structure, flavonoids feature interconnected A and B aromatic rings spanning three carbon atoms [28]. The natural flavonoid spectrum is diverse, classifiable into eight distinct categories based on the hybridization level of the central carbon atom and the B-ring’s attachment site, whether at the second or third position. These classes encompass flavones, flavonols, dihydroflavonoids, dihydroflavonols, isoflavones, flavan-3-ols, anthocyanins, and chalcones, with examples such as baicalin, gambogic acid, and puerarin depicted in Figure 1 [29,30]. Flavonoids typically manifest as crystalline granules or powders, and the hue of these compounds is influenced by the quantity, positioning, and nature of the substituents on the cross-conjugated systems and chromophores present within their molecular structure. Flavonoids have demonstrated notable medicinal properties and are recognized for their potential in preventing cardiovascular and cerebrovascular diseases [31]. These bioactive compounds are capable of strengthening blood vessels, reducing cholesterol and blood lipid levels, promoting blood flow, and averting prevalent conditions such as cerebral hemorrhage, coronary heart disease, hypertension, and angina, particularly in the geriatric population.

### 2.3. Alkaloids

#### 2.3.1. Vinblastine and Its Derivatives

Chemical analysis of extracts from *Catharanthus roseus* has confirmed the presence of vinblastine (VBL, C_46_H_58_N_4_O_9_) and vincristine, which are potent antitumor agents that have been applied in clinical settings for more than sixty years [32,33]. VBL, a complex diterpenoid indole alkaloid with a distinctive needle-like crystalline structure (as shown in Figure 1), exhibits low thermal stability and is soluble in organic solvents like methanol, acetone, and ethyl acetate but not in water or petroleum ether [34,35,36]. VBL is sensitive to light, and it decomposes easily and requires protective measures. The rarity of this valuable drug is partly attributed to the complexities involved in its synthesis and extraction.

VBL is known for its myelosuppressive toxicity and is constituted from the upper verapamil and lower vindoline moieties [37]. Conversely, vincristine is distinguished by its neurological toxicity, minimal myelosuppressive effects, and a more potent inhibitory action on tumor transplants due to the oxidation of the 1-N-methyl group on the vindoline component [38]. The slight structural variation between the two drugs has garnered considerable research attention due to its significant impact on their antitumor efficacy and toxicity profiles. Advances in chemical modifications of VBL have spurred the commercialization of more potent and less toxic agents, such as Vindseine, Vinorebline, and Vinflunine [39]. Nonetheless, the clinical use of VBL and its derivatives is constrained by issues like peripheral neurotoxicity, myelosuppression, and the emergence of P-glycoprotein (Pgp)-mediated resistance in advanced clinical stages [40]. Addressing these challenges to reduce toxicity and enhance resistance remains a critical endeavor in oncology.

#### 2.3.2. Camptothecin and Its Derivatives

In 1966, American chemists Wall and Wani first identified a pentacyclic compound known as Camptothecin (CPT, C_20_H_16_N_2_O_4_, Figure 1) [41]. CPT is characterized by its complex structure, which includes a pyridinone ring (referred to as ring D), pyrrole rings (comprising A, B, and C rings), and a distinctive six-membered *α*-hydroxylactone ring (ring E), as depicted in Figure 1. The α-hydroxylactone ring, with its asymmetric center and a 20 S configuration, along with asymmetric hydroxyl groups, is a key structural feature that endows CPT with its significant antitumor capabilities [42,43]. While CPT and its derivatives are generally insoluble in water, they exhibit good solubility in polar aprotic solvents such as methanol and dimethyl sulfoxide. Furthermore, CPT solutions emit a violet-blue fluorescence under ultraviolet light, and the ring-closing lactone form is particularly influential in determining its biological activity [44]. CPT exhibits its antitumor activity by inhibiting topoisomerase I, an enzyme essential for DNA replication. It stabilizes the enzyme-DNA cleavage complex, which leads to DNA strand breaks and subsequently inhibits cancer cell replication [45].

#### 2.3.3. Berberine

Berberine (BBR, C_20_H_18_NO_4_), an isoquinoline alkaloid depicted in Figure 1, is extensively studied for its medicinal properties. It is derived from *Coptis chinensis*, which belongs to the *Ranunculaceae* plant family [46]. Predominantly occurring as a quaternary ammonium salt in nature, BBR is found in the roots, stems, and leaves of plants from the *Berberidaceae*, *Loganiaceae*, and *Ranunculaceae* families. Although BBR can be synthesized in large quantities, its solubility in organic solvents is restricted; it is more soluble in cold water and ethanol, with solubility increasing as temperature rises [47,48]. Pure BBR presents as yellow needle-like crystals with a melting point of around 145 °C and displays yellow fluorescence under ultraviolet light [49]. The alkaloid’s diverse biological and pharmacological activities have garnered significant research interest and recent studies have highlighted its cardioprotective and neuroprotective effects, along with its antibacterial and anti-inflammatory capabilities [50,51].

#### 2.3.4. Evodiamine

Evodiamine (EVO, C_19_H_17_N_3_O) is an indole alkaloid derived from *Evodiamine rutaecarpa*, which is revered as a traditional medicinal plant. It is characterized by a unique pentacyclic structure that incorporates three nitrogen atoms (as illustrated in Figure 1) [52,53]. EVO has demonstrated a spectrum of pharmacological properties. These include analgesic, antitumor, antibacterial, and metabolic regulation capabilities. Furthermore, EVO has been recognized for its diverse biological activities, such as antithrombotic and vasodilatory actions, as well as anti-inflammatory, anti-obesity, thermoregulatory, and cardiovascular protective effects [54,55]. The broad spectrum of EVO’s activities suggests its potential across a range of therapeutic applications, from cancer and inflammatory conditions to obesity and cardiovascular diseases [56,57].

### 2.4. Polyphenols

Polyphenols constitute a varied class of natural compounds abundantly found throughout the plant kingdom, renowned for their robust antioxidant and anti-inflammatory capabilities [58,59]. Characterized by the presence of one or more phenolic rings, these bioactive substances are synthesized within plants via the shikimate and/or polyketide pathways [60].

#### 2.4.1. Curcumin

Curcumin (C_21_H_20_O_6_, Figure 1), a yellowish polyphenol obtained from the rhizome of *Curcuma longa* plants in the *Zingiberaceae* family, is used in TCMs systems to treat a range of ailments, including arthritis, stomach ulcers, dysentery, sprains, and skin infections [61]. It has shown significant antitumor activity in liver, stomach, and prostate cancers, and it showed promising results as a treatment for brain diseases, cholesterol, and endothelial dysfunction [62,63]. Additionally, curcumin is a low cytotoxicity drug and an effective anti-inflammatory and antiviral agent.

#### 2.4.2. Resveratrol

Resveratrol (C_14_H_12_O_3_), chemically known as 3,4,5-trihydroxy-trans-stilbene, was initially isolated from the roots of *Veratrum puberulum* Loes and has since been identified in more than 70 plant species. This natural polyphenolic compound is especially abundant in grapes, peanuts, *Polygonum cuspidatum*, and its initial source, *Veratrum puberulum* Loes. Purified resveratrol forms colorless needle-like crystals that are poorly soluble in water but readily dissolve in organic solvents such as acetone and ethanol. Resveratrol can exist in both cis and trans configurations; the trans isomer is the major form, while the cis isomer forms from the trans isomer under light exposure, as depicted in Figure 1 [64,65]. A wealth of research has revealed resveratrol’s diverse biological activities, including its antioxidant and anti-inflammatory properties, anticancer effects, as well as cardiovascular protection, neuroprotection, osteoporosis treatment, and antiviral effects [66,67].

#### 2.4.3. Rosmarinic Acid

Rosmarinic acid (RA, C_18_H_16_O_8_) is a naturally occurring polyphenolic compound predominantly found in plants belonging to the *Lamiaceae* and *Boraginaceae* families [68,69]. Initially identified and characterized in 1958 by Italian scientists, RA was named after its botanical origin, *Rosmarinus officinalis*. The molecular structure of RA consists of an ester linkage resulting from the condensation of caffeic acid and 3,4-dihydroxyphenyllactic acid, as shown in Figure 1. An extensive array of research has underscored the multifaceted biological activities of RA, encompassing antioxidant, anti-inflammatory, antiviral, and antithrombotic properties [70,71,72]. These activities are largely attributed to RA’s distinctive chemical composition, which includes multiple phenolic groups that enable it to neutralize free radicals and mitigate oxidative stress. Furthermore, RA has been shown to influence critical signaling pathways and gene expression associated with inflammation, thrombosis, and viral infections, underscoring its promise as a therapeutic target for innovative treatment modalities.

#### 2.4.4. Anthocyanins

Anthocyanins (ANCs) are water-soluble pigments predominantly present in fruits and vegetables, responsible for a spectrum of colors ranging from blue and purple to red. They are the principal polyphenolic constituents in red cabbage and other color-rich plant and vegetable extracts, known for their natural antioxidant properties [73,74]. ANCs are characterized as 2-phenylbenzopyranonium cation derivatives, featuring rings A, B, and the heterocyclic C ring, with variations occurring in the groups attached to ring B (as shown in Figure 1). To date, over 250 distinct naturally occurring anthocyanins have been identified, with six predominant types found across plants: pelargonidin, cyanidin, delphinidin, peonidin, malvidin, and petunidin [75]. ANCs are associated with a wide array of biological activities, such as antitumor, anti-inflammatory, and antioxidant actions, as well as memory enhancement, blood pressure reduction, cognitive improvement, promotion of radiation resistance, and anti-atherosclerotic effects [76,77].

## 3. Polymer Nanocarriers in Delivery of Active Ingredients from TCMs

**Figure 2:**
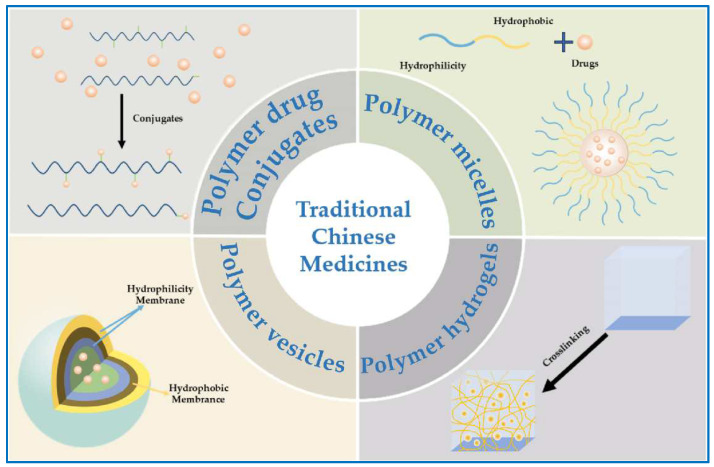
Illustration of polymer nanocarriers in the delivery of active ingredients from TCMs.

Innovative drug delivery systems frequently leverage polymer carrier materials, which are pivotal in driving advancements in pharmaceutical formulation, smart manufacturing, and novel drug development. Polymer nanocarriers offer diverse drug loading techniques, such as covalent attachment to create polymer–drug conjugates, the encapsulation of drugs within polymer micelles or vesicles, and the dispersion of drugs in polymer gels (Figure 2) [78,79,80,81]. These carriers are distinguished by their precise biocompatibility, minimal toxicity and antigenicity, tunable drug loading and release profiles, ability to target specific cellular or subcellular sites, enhancement of drug efficacy, reduction in side effects, and applicability to a broad range of drug types including small molecules, proteins, peptides, and nucleic acids [82]. Several commercially available polymer carriers include Risperdal Consta^®^, Trelstar^®^, Sandostatin LAR^®^, Eligard^®^, Genexol^®^, Nanoxel^®^, Somatuline Autogel^®^, etc. [83].

### 3.1. Polymer Micelles

**Figure 3:**
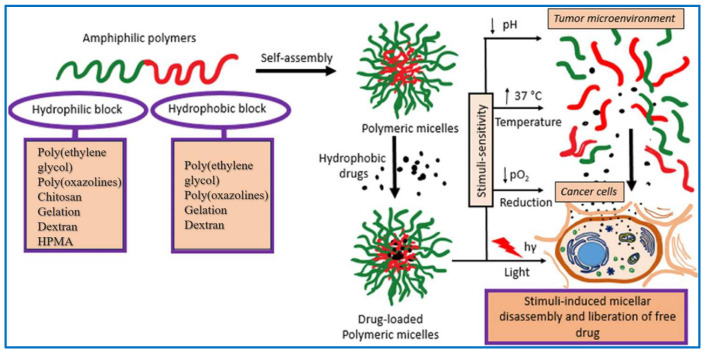
Schematic illustration of the formation and application of polymer micelles [92]. Reproduced with permission from reference [92].

Polymer micelles (PMs) have emerged as promising nanocarriers due to their ability to self-assemble from amphiphilic polymers, forming core–shell structures that can encapsulate hydrophobic drugs [84,85,86,87]. PMs feature a hydrophobic core capable of encapsulating lipophilic chemotherapeutic agents like paclitaxel (PTX), thereby enhancing their solubility [88]. Concurrently, the micelles’ hydrophilic shell forms a hydration layer that acts as a protective barrier, reducing protein adsorption and avoiding rapid clearance by the reticuloendothelial system, which contributes to a prolonged drug half-life. PMs generally have a small and uniform particle size between 10 and 100 nm, a characteristic that can be tailored by adjusting the length of the hydrophilic blocks [89,90,91]. A case in point is Genexol^®^ PMs, a clinically approved formulation that employs methoxy polyethylene glycol-poly(D, L-lactic acid) (mPEG-*b*-PDLLA) amphiphilic block copolymers to create spherical micelles for PTX encapsulation, presenting a promising therapeutic option for ovarian and non-small-cell lung cancers (Figure 3) [92].

Rytting et al. engineered biodegradable and biocompatible nanoparticles from PEGylated poly(lactide-glycolic acid) (PLGA) and successfully encapsulated digoxin with high encapsulation efficiency via a modified solvent displacement method [93]. The PMs could release digoxin uniformly within 48 h and exhibited a 2.5-fold increase in cellular permeability compared to the free drug. Remarkably, PEGylated PLGA PMs showed no cytotoxic effect on trophoblast cells at concentrations up to 200 μg/mL. Furthermore, these PMs can shield digoxin from P-gp-mediated efflux in the placental trophoblast layer and this protective measure enhances the mother to fetus transfer, an advantageous attribute in refining fetal pharmacotherapy. In another study, Zhai et al. developed an innovative redox-responsive nanoparticle system for the targeted delivery of docetaxel (DTX) by incorporating cystamine (Cys), a sulfated glycosaminogly with the ability of tumor targeting, with disulfide bonds to form a Cys-DTX conjugate, which was then used to create redox-responsive Cys-DTX/CS-ss-DTX nanoparticles via the self-assembly of amphiphilic polymer [94]. Cys-DTX/CS-ss-DTX facilitated enhanced DTX release in reductive environments with a 30% cumulative release of DTX in pH 7.4 after 96 h, while the cumulative release of DTX in pH 7.4 after 96 h was 50%, which could be due to the improved solubility of Cys-DTX under more acidic conditions. Interestingly, the release kinetics were accelerated by the addition of 10 mM DTT due to the presence of a redox-sensitive disulfide linkage in the PMs. In addition, the PMs also exhibited improved tumor tissue permeability and increased cytotoxicity while minimizing side effects. These promising results indicate that the Cys-DTX/CS-ss-DTX nanoparticle system could be a significant advancement for cancer chemotherapy in the future.

**Figure 4:**
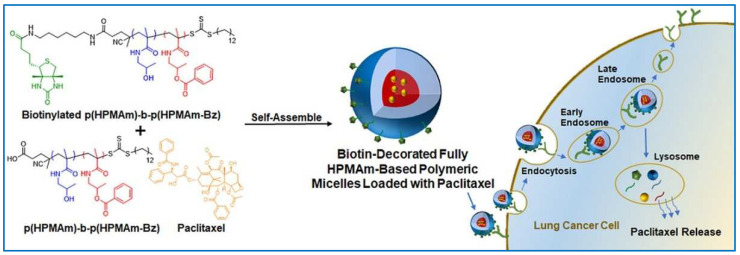
The structure of biotin-decorated amphiphilic block copolymer and its application in the delivery of hydrophobic anticancer drugs of Paclitaxel [96]. Reproduced with permission from reference [96].

Ni et al. created spherical PMs with diameters consistently between 110 and 180 nm using cross-linked xanthan gum, which demonstrated responsiveness to reductive conditions, thereby enhancing drug loading efficiency and preventing drug leakage. In vitro studies indicated that drug release from these PMs could be modulated by pH and reductive conditions, simulating the tumor microenvironment [95]. The biocompatibility of these nanoparticles positions them as promising candidates for targeted anticancer drug delivery. Hennink et al. reported the synthesis amphiphilic block copolymers compromising poly(N-2-hydroxypropylmethacrylamide) and poly (N-2-benzoyloxypropyl methacrylamide) by reversible addition–fragmentation chain-transfer polymerization using a biotin-functionalized chain transfer agent, 4-cyano-4-[(dodecylsulfanylthiocarbonyl)-sulfanyl]pentanoic acid (Figure 4) [96]. In aqueous solutions, the copolymers spontaneously organized into micelles measuring 40–90 nm in diameter, a size that increased in direct proportion to the length of the hydrophobic segments within the polymer chains. The PTX-loaded PMs, formulated using a polymer of 22.1 kDa molecular weight, ranged in size from 61 to 70 nm and achieved a peak loading capacity of approximately 10% by weight. A549 lung cancer cells, characterized by an overexpression of the biotin receptor, demonstrated a heightened internalization of biotin-modified micelles compared to their non-targeted counterparts. In stark contrast, HEK293 human embryonic kidney cells, deficient in the biotin receptor, showed minimal uptake of both micelle variants. Their findings clearly demonstrated that PMs are good candidates for hydrophobic drug delivery.

### 3.2. Polymer Vesicles

Polymeric vesicles are a class of self-assembling hollow spheres in solution, structurally analogous to liposomes, and are constructed from amphiphilic block copolymers [97,98,99,100]. Specifically, the hydrophobic segments of the block copolymers form a membrane layer in the middle of the vesicle, while the hydrophilic segments form brush-like structures on the inside and outside of the membrane [101,102,103]. This unique structure creates a hydrophilic inner cavity and hydrophobic membrane, making it an ideal environment for encapsulating both hydrophilic and hydrophobic cargo. By tuning the structure, type, and molecular weight of the block copolymers, vesicles can be engineered to possess specific characteristics and functions to target different diseases. Compared to lipids, block copolymers offer greater synthetic versatility, enabling researchers to design and tailor vesicles for a wide range of applications, including drug delivery, food industry, cosmetics, and medical diagnostics [104,105,106].

Polymeric vesicles have emerged as a highly desirable platform for the encapsulation of TCMs due to their unique structure, which efficiently accommodates both hydrophilic and hydrophobic drugs [107,108]. A common strategy for fabricating drug-loaded vesicles is the direct incorporation of the drug during the vesicle formation process. Hubbell et al. introduced an innovative ‘direct hydration’ technique, which combines solvent dispersion with homopolymer addition to encapsulate biomacromolecules [109]. Zhou et al. successfully loaded hydroxychloroquine (HCQ), a hydrophilic anticancer drug, and tunicamycin (Tuni), a hydrophobic anticancer drug, into the lumen and membrane layer of polymer vesicles composed of PEG-*b*-poly(propylene thioether) (PEG-*b*-PPS) [110]. These drug-loaded vesicles can selectively accumulate in tumor tissue through the enhanced permeability and retention effect and enter cells via endocytosis. The concurrent delivery of HCQ and Tuni induced endoplasmic reticulum (ER) stress and obstructed autophagic flux, leading to significant antitumor effects and reduced metastasis. In another study, Discher et al. co-encapsulated PTX and doxorubicin (DOX) within PEG-PLA/PEG-PBD hybrid vesicles [111]. Administered intravenously to mice bearing pre-implanted tumors, these vesicles demonstrated a higher tolerated dose compared to the individual administration of DOX and PTX monotherapies. Moreover, the vesicles showed superior efficacy in inducing tumor tissue necrosis compared to monotherapies, underscoring their potential as a promising multi-drug delivery system.

**Figure 5:**
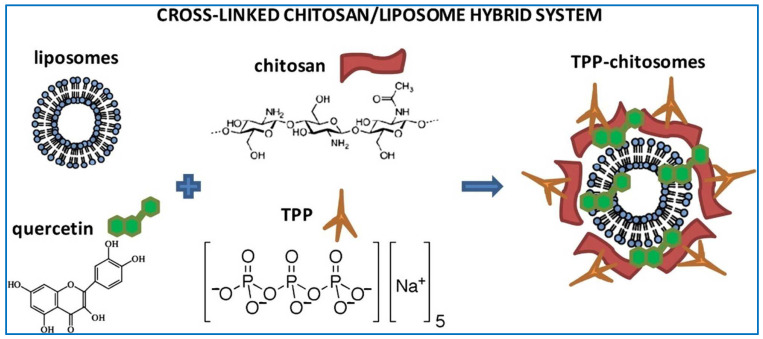
Illustration of the structure of the chitosan/liposome hybrid system in the delivery of quercetin [115]. Reproduced with permission from reference [115].

Liposomes have many advantages, including high biocompatibility, low cytotoxicity, chemical versatility for hydrophilic, amphiphilic, and lipophilic compounds, and facile modulation of pharmacokinetic properties by compositional mediation, and they are widely used in the delivery of TCMs [112,113,114]. Caddeo et al. designed a novel hybrid system containing biocompatible, biodegradable, and biorenewable chitosan and liposomes to improve the bioavailability of hydrophobic and instable quercetin and optimize its release behavior in the intestine [115]. In the preparation of polymer vesicles, liposomes and quercetin were first formed into vesicles and then coated with cross-linked chitosan to improve the effectiveness and feasibility, as illustrated in Figure 5. Remarkably, the hybrid polymer vesicle system exhibited a desirable particle size distribution, with diameters consistently under 180 nm, coupled with an impressive encapsulation efficiency exceeding 91%. In vitro experimentation revealed that the release of quercetin was pH-dependent, with a preference for alkaline conditions, and appeared to be predominantly regulated by the release of the drug through the hybrid polymer vesicles. The results suggest that the hybrid system of liposomes and polymer vesicles may be a good candidate for delivering TCMs to treat inflammatory intestine disease.

**Figure 6:**
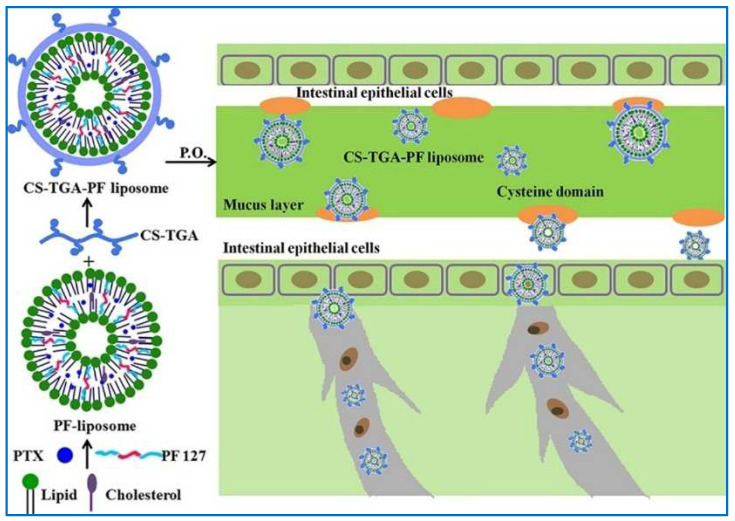
Schematic illustration of the forming process, intestinal mucus adhesion, and penetration mechanisms of PTX-loaded CS-TGA-PF liposomes [116]. Reproduced with permission from reference [116].

In order to improve the solubility and intestinal permeability for the oral PTX-based nanocarrier delivery system, Liu et al. synthesized novel hybrid polymer vesicles with the mucus adhesion- and penetration-functionalized chitosan-thioglycolic acid-Pluronic F127 (CS-TGA-PF) liposome (Figure 6) [116]. Well-designed PTX-loaded CS-TGA-PF liposomal formulation exhibited a particle diameter of 121.4 nm and a zeta potential of 50.2 mV. These CS-TGA-PF liposomes displayed enhanced stability compared to their unmodified counterparts and exhibited a controlled-release profile of PTX when incubated in both simulated gastric and intestinal fluids. It was observed that a three-fold amount of mucin was absorbed by the hybrid polymer vesicle compared to the unmodified liposomes. Furthermore, CS-TGA-PF also exhibited enhanced GI mucosa uptake and intestine drug absorption.

### 3.3. Polymer Hydrogels

Polymeric hydrogel-based drug delivery systems are gaining recognition as an effective approach for the targeted and sustained release of TCMs due to their remarkable biocompatibility, tunable physicochemical properties, and ability to encapsulate drugs with varying solubility profiles [117,118,119,120,121]. As three-dimensional network structures, hydrogels possess the unique ability to absorb and retain significant volumes of water or biological fluids without compromising their structural stability. These characteristics render hydrogels well-suited for controlled and sustained drug delivery applications.

A multitude of studies have delved into the potential of polymer hydrogel systems for the delivery of TCMs [122]. For instance, Li et al. reported the preparation of catechol-modified chitosan-based hydrogels to overcome the lack of adhesion features in conventional hydrogels and evaluated the delivery of *Panax notoginseng* to wound sites [123]. The cross-linked hydrogels were covalently bonded based on the crosslinking reaction of *o*-diquinone from the oxidation product of catechol and the amine of chitosan, or the coordination between catechol and Fe^3+^. It was found that the maximum bonding strength of the hydrogel could reach 9.72 kPa. Remarkably, 1 g of the produced hydrogel could load 8.61 mg of *Panax notoginseng* and the cumulative release rate after 60 h reached 91.76% and 67.16%, due to the multi-dimensional porous structure. Furthermore, the prepared hydrogel could enhance the wound healing rate, reduce infections, and stimulate capillary formation, inflammatory exudate absorption, and granulation tissue formation, thereby promoting effective wound healing in mice. Similarly, the cross-linked hydrogel based on catechol-modified chitosan was used to load flavones of Resina Draconis for infectious wound healing [124]. The polymeric hydrogel loaded with flavones of Resina Draconis showed slow, sustained cumulative release rates of 71.9% after 60 h at pH 6.8 and performed complete wound healing after 7 d with a healing rate of 94.5%. These results demonstrated that the delivery of TCMs with chitosan-based hydrogels provides a plausible route for infection treatment and wound healing.

**Figure 7:**
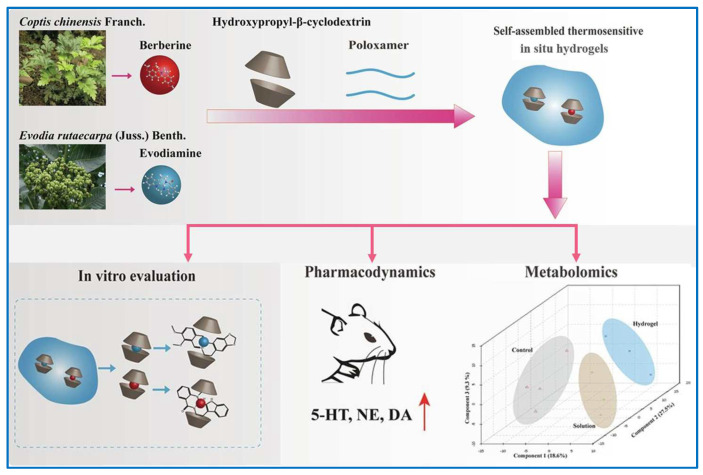
Schematic illustration of drug delivery hydrogel formation and its application in intranasal administration as an antidepressant [126]. Reproduced with permission from reference [126].

Crafting innovative approaches to transport a higher concentration of antidepressants across the blood–brain barrier is advantageous for addressing neurological disorders, with a particular emphasis on the treatment of depression. Cui et al. developed a novel route by delivering BBR intranasally to improve its antidepressant-like performance [125]. BBR was loaded with high efficiency (22.86%) by the in situ-formed thermosensitive (30 °C) hydrogels comprising hydroxylpropyl-*β*-cyclodextrin and poloxamers P407 and P188 due to hydrogen bonding and hydrophobic interaction. A prolonged release profile of BBR was noted, beginning after a 6 h period, with the total cumulative release rate averaging 83.29 ± 3.98%. Moreover, the intranasal administration of hydrogel formulated with BBR demonstrates superior bioavailability (110 times to oral) and heightened antidepressant efficacy, even at a reduced dosage compared to intragastric administration, which could be attributed to a distinctive mechanism that rectifies mitochondrial dysfunction and normalizes irregularities in phospholipid and sphingolipid metabolism. On the basis of the above results, the same group then further improved the antidepressant efficacy by intranasal administration, and BBR and EVO were co-administrated in similar thermosensitive hydrogels (Figure 7) [126]. Remarkably, the intranasal administration hydrogel formulated with BBR and EVO demonstrates superior bioavailability (132 times that of oral) and heightened antidepressant efficacy, even at a reduced dosage compared to intragastric administration. Consequently, serving as a co-delivery drug system, the hydrogels, when administered intranasally, offer a well-regulated release profile and enhanced bioavailability, thereby presenting a noninvasive therapeutic approach for the clinical management of depression.

Cui et al. reported an innovative approach to bone reconstruction, particularly in the context of osteoporotic fractures [127]. The study focuses on the development of a multifunctional drug delivery system that integrates adhesive liposomes (A-LIP) with an injectable hydrogel to enhance local bone reconstruction by hybrid SH-PEG and Ag^+^ ions hydrogel with octadecylamine modified liposomes loaded with bone morphogenetic protein-2, lecithin, and cholesterol, giving the hydrogel antibacterial, bone growth, and self-healing properties. Chen et al. researched the development of a lipo-hydrogel, a hybrid material that combines the properties of liposomes with those of hydrogels prepared by UV initiated polymerization of methacrylate-modified gelatin under mild conditions [128]. At the same time, different types of liposomes carrying drugs such as deferoxamine, PTX, bovine serum albumin, and bone morphogenetic protein-2 were incorporated into the micro-cross-linking double-network structure, which not only enhances the mechanical strength of the hydrogel but also enables the controlled release of various drugs that are crucial for bone regeneration. This system ensures the early release of hydrophilic drugs like deferoxamine, which can promote angiogenesis, followed by the mid-term release of bioactive macromolecules, such as bovine serum albumin and bone morphogenetic protein-2, and the long-term release of lipophilic drugs like PTX. This strategic release pattern is tailored to support the complex biological processes involved in bone healing, including vascularization and osteogenesis. Polymeric hydrogel-based drug delivery systems offer a promising avenue for the targeted, sustained, and controlled delivery of AITCMs. These systems have shown potential to increase drug bioavailability, reduce side effects, and enhance therapeutic efficacy. Continued research is anticipated to refine these systems for clinical applications and contribute significantly to the evolution of innovative TCM formulations.

### 3.4. Polymer Drug Conjugates

It is important to note that PMs can sometimes impede drug loading due to intermolecular hydrophobic interactions, which may cause drug aggregation, decrease loading capacity, and lead to unpredictable drug ratios. To address this, one strategy is to replace the non-covalent donor–receptor interactions between the polymer and the drug with covalent bonds, creating polymer–drug conjugates [129,130,131,132]. These conjugates are a category of therapeutic agents where a bioactive drug is covalently attached to a polymeric carrier molecule, which is instrumental in the pharmaceutical field for enhancing the drug’s solubility, stability, and bioavailability while minimizing its toxicity and immunogenicity [133,134,135]. The synthesis of PDCs can be achieved through chemical or biological methods, resulting in a single macromolecule with a defined molecular weight and release profile [136,137]. This method ensures precise control over the drug-to-polymer ratio, pharmacokinetics, and pharmacodynamics, thereby optimizing drug efficacy and safety. Owing to their customizable properties, PDCs have demonstrated significant potential in treating a variety of diseases, including cancer, inflammatory conditions, and autoimmune diseases. Furthermore, acknowledging that acidic environmental degradation and redox reactions are the primary pathways for the release of covalently bound TCM components in vivo, the majority of PDCs described in the literature rely on the use of ester and disulfide linkages. These conjugation strategies, which are pivotal for the controlled release of TCMs, will be detailed in the subsequent sections.

**Figure 8:**
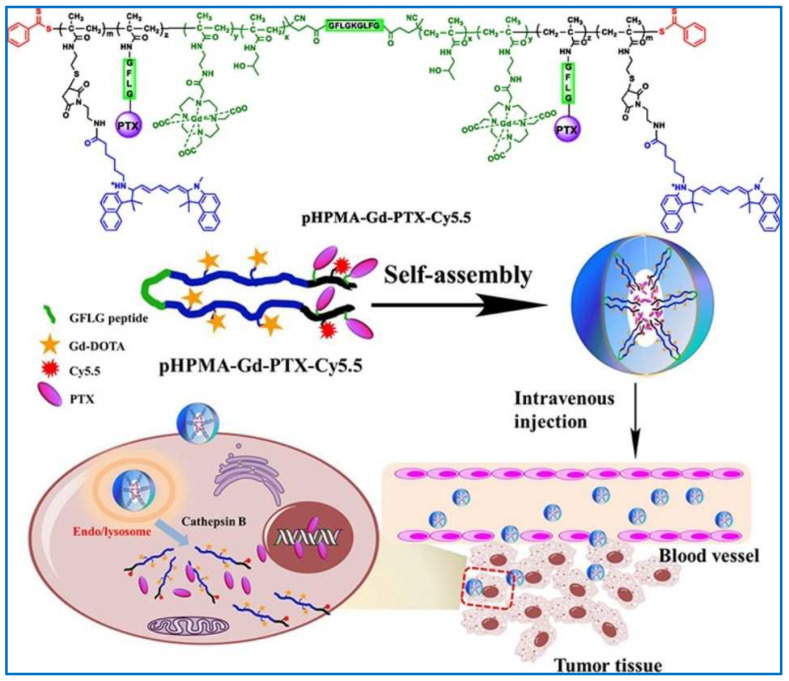
Illustration of the application of polymer–drug conjugates [138]. Reproduced with permission from reference [138].

Luo et al. developed a novel amphiphilic biodegradable complex of N-(2-hydroxypropyl methyl) acrylamide (HPMA) copolymer conjugated with gadolinium, paclitaxel, and Cyanine5.5 (pHPMA-Gd-PTX-Cy5.5), based on paclitaxel-Cyanine5.5. Their findings validated the efficacy of these conjugate-based prodrugs, which demonstrated inhibition of proliferation and reduced apoptosis in 4T1 murine breast cancer cells (Figure 8) [138]. Wei et al. created monomethoxy-PEG-*b*-poly(lactide) (mPEG-PLA) conjugated with docetaxel (DTX) through an ester linker (DTX-PM) due the pronounced biodegradability and biocompatibility of mPEG-PLA and effective oral cancer treatment. The DTX-PM demonstrated a prolonged drug release profile over a period of 160 h, with a slightly enhanced release rate under acidic conditions due to the well-defined ester linkage. Therefore, at the 24 h mark, the free drug showed a potential antitumor impact, but by the conclusion of a 48 h incubation, DTX-PM displayed a more pronounced antitumor efficacy [139].

Koul et al. synthesized multiblock PDCs featuring trastuzumab and folic acid, linked via an ester bond between folic acid (FA) and the polymer backbone hydroxyl groups [140]. These prodrugs showed an impressive drug loading capacity of 22% and induced 80% apoptosis in MCF-7 breast cancer cells, compared to only 20% with the free drug. In another recent study, Hong et al. developed *β*-cyclodextrin-polycaprolactone block copolymers and functionalized them with FA to fabricate curcumin-loaded nanoparticles (FA-CUR-NPs) via the emulsion evaporation technique [141]. Notably, under the acidic conditions mimicking the tumor microenvironment (pH 6.4), the FA-CUR-NPs exhibited a curcumin release rate that was threefold higher compared to the rate observed under physiological pH conditions (pH 7.4). When administered orally, FA-CUR-NPs were found to significantly reduce tumor volume by three times compared to free curcumin and by two times compared to non-targeted CUR-NPs after a 30 d treatment period in mice. These findings underscore the enhanced therapeutic potential of FA-CUR-NPs in vivo and highlight the efficacy of FA as a tumor-targeting moiety, facilitating improved cellular uptake. Fru et al. designed PDCs of polyethylene glycol-betulinic acid (PEG-BA) and assessed their cell death, anti-inflammatory, and antioxidant activities in pancreatic cancer [142]. By arresting MIA-PaCa-2 cells in the Sub-G1 phase, evaluating NF*κ*B/p65 protein expression and the proapoptotic of *TNF* (23.72 ± 1.03) and *CASPASE 3* genes, their results highlighted the superior anticancer activity of the PEG-BA prodrugs, which triggered both the intrinsic and extrinsic pathways of the apoptosis duo to improve the reduction in hydroperoxide levels compared with BA-only.

Peripheral nerve injury poses a formidable clinical challenge, imposing significant burdens on affected individuals, characterized by its high prevalence and the inadequacy of current therapeutic strategies. Lu et al. reported the incorporation of gastrodin, a pharmacologically active constituent with excellent neuroprotective and anti-inflammatory properties derived from the plant *Gastrodia elata*., into the polyurethane backbone as the chain extender in a weight ratio of 1 and 5 wt%. They successfully facilitated the recuperation of sciatic nerve functionality by cultivating a conducive microenvironment for nerve regeneration with gastrodin-modified polyurethane. This was primarily achieved by modulating the expression of iNOS and TNF-*α*, thereby leveraging the bioactivity of gastrodin. Consequently, this intervention mitigated the inflammatory reactions typically induced by nerve guidance fibers, which can impede the nerve repair process. Collectively, these efforts enhanced the functionality of Schwann cells and promoted the formation of nerve fibers in conjunction with angiogenesis [143].

**Figure 9:**
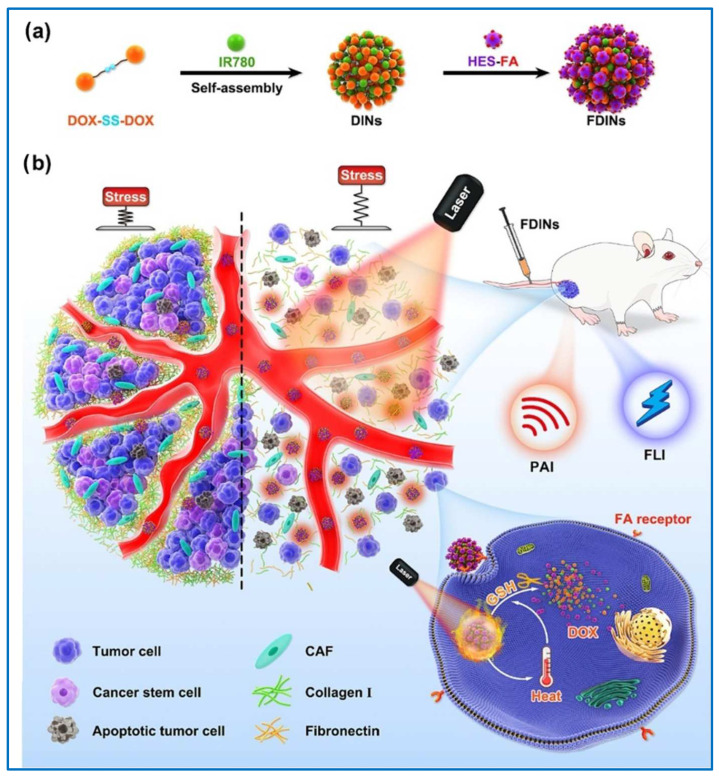
(**a**). FDINs’ preparation. (**b**). FDIN-mediated tumor mechanical microenvironment modulation for enhanced cancer stem cell elimination and antitumor efficacy [147]. Reproduced with permission from reference [147].

Self-immolative linkers equipped with a disulfide moiety, known as SS-SILs, constitute an efficacious strategy for the intracellular delivery of drugs via PDCs [144]. The elevated concentrations of the reducing agent glutathione (GSH) within the endosomal environment, coupled with the activity of a specific reducing enzyme such as gamma-interferon–inducible lysosomal thiol reductase, facilitate the release of the drug from these SS-SILs, which is particularly advantageous for targeted drug delivery in the intracellular milieu [145,146]. Li et al. reported the synthesis of a nanomedicine (FDINs) with hydroxyethyl starch-FA (HES-FA) conjugates and its application in stabilized theranostic nanoparticles, where the nanoparticles were loaded with a disulfide linked DOX dimeric prodrug (DOX-SS-DOX) and a photothermal agent, IR780 iodide (Figure 9) [147]. The FDINs were designed to actively target triple-negative breast cancer 4T1 tumor tissues and it achieved reduction-responsive drug release due to the high GSH concentration in cancer cells and cancer stem cells (CSCs). Their findings demonstrated that FDINs potently eliminated CSCs by disrupting their unique niche and consuming intracellular GSH, which is crucial for their survival and stemness due to the sensitive DOX-SS-DOX and HES-FA.

Significantly, the stability of the disulfide linker can be readily adjusted by incorporating different spacers between the disulfide bond and the therapeutic agent, such as methyl-disulfide or ethyldisulfide (Et-SS). Nonetheless, the rate at which the linker undergoes self-immolation through the intramolecular cyclization mechanism for drug release may decelerate under acidic conditions, as typically found in endosomal and lysosomal compartments and the tumor microenvironment [148,149]. This challenge has stimulated the exploration of alternative spacers in the design of SS-SILs, with phenyldisulfide linkers (Ph-SS) emerging as a promising candidate. Vicent et al. synthesized various peptide-conjugated drugs with Ph-SS linkage and evaluated the influence of the chemical group of the drug utilized for conjugation on drug-release behaviors [150]. Their results showed that fasudil has a significantly higher release function in Ph-SS-linked peptides than in Et-SS-linked peptides in MBA-MD-231 in vitro models, which highlight the influence of drug structural features on release kinetics. Table 1 was used to facilitate access to the relevant reference.

## 4. Conclusions and Perspective

TCMs encounter several challenges in clinical applications, such as low bioavailability, poor solubility, brief in vivo release duration, and potential side effects. This review provides an overview of prevalent TCMs in this domain, encompassing terpenoids, polyphenols, flavonoids, and alkaloids. To tackle these issues, researchers have directed significant efforts towards nanocarriers, which have garnered considerable interest due to their capacity to enhance TCMs solubility, ensure excellent stability, boost absorption, and offer controlled sustained release and targeting. Furthermore, this review compiles the primary types of polymer nanocarriers utilized in this field, including polymer micelles, vesicles, hydrogels, and drug conjugates. These nanocarriers have shown tremendous potential in amplifying the therapeutic efficacy of TCMs and mitigating the obstacles associated with their clinical use.

Although the literature abounds with studies, the integration of nanomedicine in TCM delivery is still in its infancy. The application of TCMs within nanomedicine is hindered by a limited scope, inadequate theoretical foundations, and incomplete fundamental research. The uncertainty surrounding the specific therapeutic effects of TCM components against various diseases adds to the complexity of developing efficient nanocarriers. Moreover, the incorporation of nanocarriers into nanomedicine could lead to increased toxicity and immunogenicity, along with higher production costs and unforeseen risks. Consequently, the development of novel nanocarriers necessitates definitive evidence of their clinical efficacy, stability, and cost-effectiveness. A deeper understanding of metabolic pathways is essential to ensure the safety of nanocarriers in clinical use and to inform their logical design. To propel this field forward, extensive pharmacokinetic and pharmacodynamic studies of nanocarriers loaded with TCM fractions are imperative. These studies will inform the rational design and development of potent nanocarriers for TCM delivery by elucidating drug delivery mechanisms, pharmacokinetic profiles, safety, and efficacy. With ongoing research and enhanced comprehension, the substantial potential of nanomedicine in TCM delivery can be harnessed, ultimately enhancing patient outcomes and quality of life.

Although various nanomaterial delivery systems have been utilized to overcome barriers associated with TCMs, individual nanomaterials still have inherent limitations. To address this, the creation of diverse nanomaterial hybrids through thoughtful design and effective strategies is anticipated to surpass the limitations of current TCM carriers and expand their biomedical applications. Polymer/inorganic nanohybrids represent nanoscale composites with distinctive functionalities, achieved by integrating polymer materials with inorganic nanoparticles through specific techniques [151,152]. During the formation of these nanohybrids, interactive forces between the polymer and inorganic phases, such as electrostatic interactions and hydrogen bonding, naturally occur. Consequently, these polymer/inorganic nanohybrids harness the combined benefits of polymer materials and inorganic nanoparticles, including superior stability, safety, and smart environmental responsiveness in drug release. Moreover, the morphology, size, and functionality of nanohybrids can be selectively tailored by modulating the ratio of polymer to inorganic components, thereby overcoming many of the individual limitations encountered during application. However, there is a scarcity of the literature regarding the application of polymer/inorganic nanohybrids in TCMs, and there is an urgent need to explore this avenue given the potential benefits of polymer/inorganic nanohybrids.

## Figures and Tables

**Table 1:** Summary of the delivery of active ingredients from TCMs with polymeric nanocarriers.

Nanocarriers	Materials	TCMs	Advantages	Reference
PMs	Carboxymethyl chitosan-rhein	PTX	A drug loading capacity of 35.46 ± 1.07%; improved absorption of PTX in the intestine with negligible intestinal villi injury	[88]
	PEGylated PLGA polymer (Resomer^®^ RGPd50105 and RGPd5055)	Digoxin	Cross BeWo b30 cell monolayers easily; high encapsulation efficiency and sustained release; increased the permeability of digoxin	[93]
	Chondroitin sulfate;	DTX	High permeability and cytotoxicity of Cys-DTX prodrug, targeting transportation of encapsulated redox-responsive Cys-DTX prodrug; improved permeability in tumor tissues, enhanced cytotoxicity, and decreased side effects	[94]
	Cystamine; Xanthan gum	Resveratrol	Good redox responsiveness; biocompatible; controlled in vitro drug release similar to the internal environment of tumor cells	[95]
	Biotin modified p(HPMAm)-*b*-p(HPMAm-Bz)	PTX	Controlled structure of polymers and PM size; high efficiency in A549 lung cancer cells overexpressing and pretty low internalization; stronger cytotoxicity in A549 cells	[96]
	Hyaluronate-chitosan-liposomes	Quercetin	High stability and skin permeation; controlled release behavior	[107]
	Carboxymethyl chitosan and chitosan (TMC)-coated liposomes	Curcumin	High stability and safety; favorable gastric acid tolerance; satisfactory biocompatibility and oral absolute bioavailability	[108]
Polymer vesicles	PEG-*b*-PPS	Hydroxychloroquine; tunicamycin	Simultaneously inducing endoplasmic reticulum stress and autophagic flux blockade; inhibiting tumor metastasis	[110]
	PEG-PLA/PEG-PBD hybrid vesicles	PTX	Thick hydrophobic membrane and an aqueous lumen to efficiently carry both hydrophobic and hydrophilic drugs; higher maximum tolerated dose; controlled drug release; two-fold higher cell death in tumors than free drug	[111]
	Chitosan and liposomes	Quercetin	Impressive encapsulation efficiency; pH-dependent release of quercetin	[115]
	PEGylated liposomes	Goniodiol	Reduced leakage and degradation of goniodiol; enhanced stability and cytotoxicity	[112]
	Phospholipid dioleoylphosphatidylcholine and liposomes	Curcumin	Pronounced solubility and stability of loaded curcumin; rapid release; high in vivo efficacy	[113]
	Chitosan-thioglycolic acid-Pluronic F127	PTX	Well-designed formulation; controlled-release profile; enhanced GI mucosa uptake and intestine drug absorption	[116]
Polymer hydrogels	Catechol-modified chitosan	*Panax notoginseng* or flavones	Enhanced bonding strength and drug loading efficiency; porous structure; sustained cumulative release rates; high wound healing rate	[123,124]
	Hydroxylpropyl-*β*-cyclodextrin and poloxamers P407 and P188	BBR and EVO	High drug loading efficiency and total cumulative release rate; thermosensitive; higher intranasal administration	[125,126]
	SH-PEG, Ag+ and liposomes	Cholesterol	Injectable hydrogel; enhanced antibacterial, bone growth, and self-healing properties	[127]
	Methacrylate-modified gelatin and liposomes	PTX	Enhanced the mechanical strength and controlled drugs’ release; enabling complex bone healing	[128]
PDCs	pHPMA-Gd-PTX-Cy5.5	PTX	Enhanced imaging capacity of the theranostic nanomedicine; residence time significantly prolonged; increased accumulation at the tumor site; inhibited proliferation and induced apoptosis of the 4T1 murine breast cancer cells	[138]
	Indole-chalcone derivatives	VBL, CPT and PTX	Facile structure design; pronounced anti-proliferative activity; wide drug-resistant variants; lower cytotoxicity to human normal cells; enhanced intracellular uptake	[135]
	mPEG-PLA	DTX	Clear spherical shape; sustained release of the drug; time-dependent anticancer effect in the squamous cancer cells; significantly higher cancer cell apoptosis in HSC-3 cancer cells; controlled the tumor progression in HSC-3 cancer cells	[139]
	*β*-Cyclodextrin-polycaprolactone and FA	Curcumin	Well-designed PDC structure; higher curcumin release rate; reduced tumor volume	[141]
	Polyethylene glycol-BA (PEG-BA)	BA	Increased NF*κ*B/p65 protein expression; comparable antioxidant potential with ascorbic acid; improved reduction in hydroperoxide levels	[142]
	Polyurethane	Gastrodin	Tunable gastrodin content; leveraging the bioactivity of gastrodin; mitigated inflammation; enhanced nerve repair process	[143]
	Hydroxyethyl starch-FA	FA	Redox-sensitive; targets triple-negative breast cancer 4T1 tumor tissues	[147]
	PEGylated-PTX and polyester	PTX	Controlled chemical structure; redox-sensitive; controlled drug release rate; excellent stability and safety	[145]

## Data Availability

The data could be provided if they are required by others.
